# Supplementary material for: Efficient candidate drug target discovery through proteogenomics in a Scottish cohort
Source: Commun Biol. 2025 Aug 29;8:1300. doi: 10.1038/s42003-025-08738-w (PMC12397405; doi:10.1038/s42003-025-08738-w)
Supplement: Supplementary file 2 — Supplementary Information [file 42003_2025_8738_MOESM2_ESM.pdf]

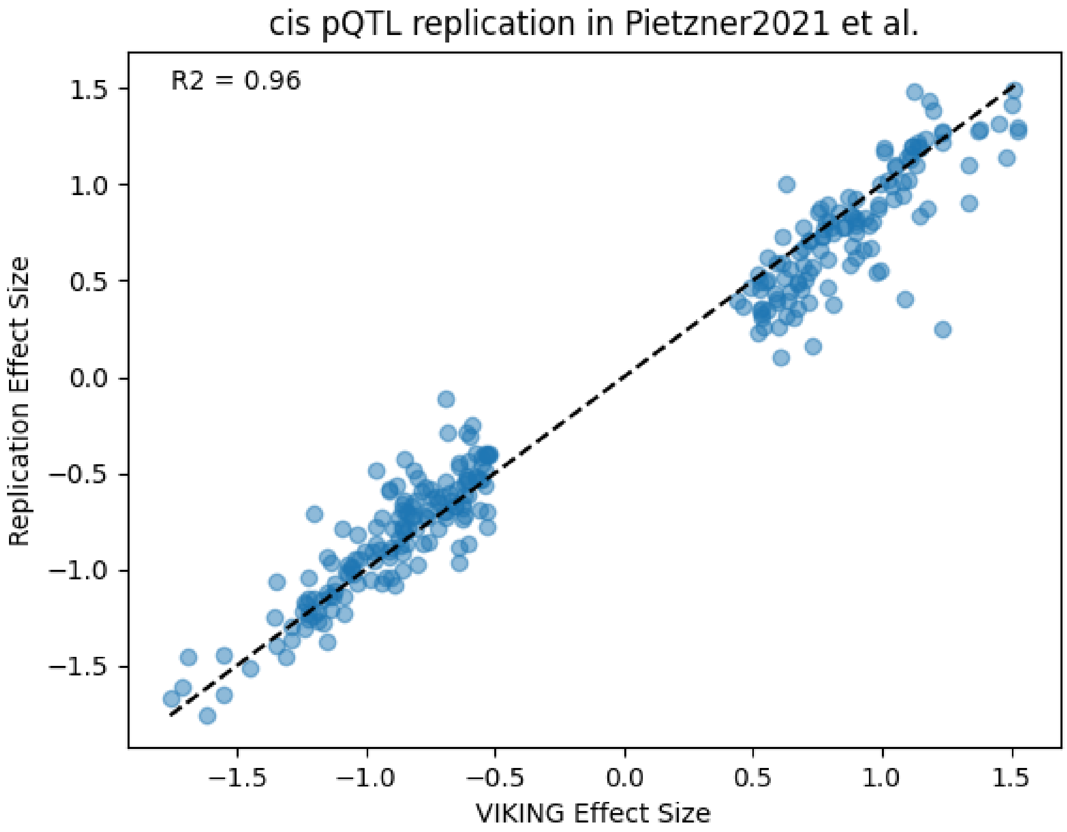

Supplementary Figure 1. Scatter Plot of *cis*-pQTL Replication. This scatter plot shows the replication of *cis*-pQTL effect sizes between the VIKING cohort and the Fenland cohort as reported in Pietzner et al. (PMID: 34648354). The x-axis represents the effect sizes from the VIKING study, while the y-axis corresponds to the replicated effect sizes from the Fenland cohort. Each point in the plot represents a matched pQTL between the two studies, and the dashed line represents the line of equality (x = y), where perfect replication would lie. The high degree of consistency in effect sizes is evident, with all pQTLs matching in directionality between the two cohorts, and an overall strong correlation of Pearson $r^2 = 0.96$, indicating excellent replication of genetic associations. This high concordance underscores the robustness of the findings and supports the reliability of the reported *cis* pQTL.

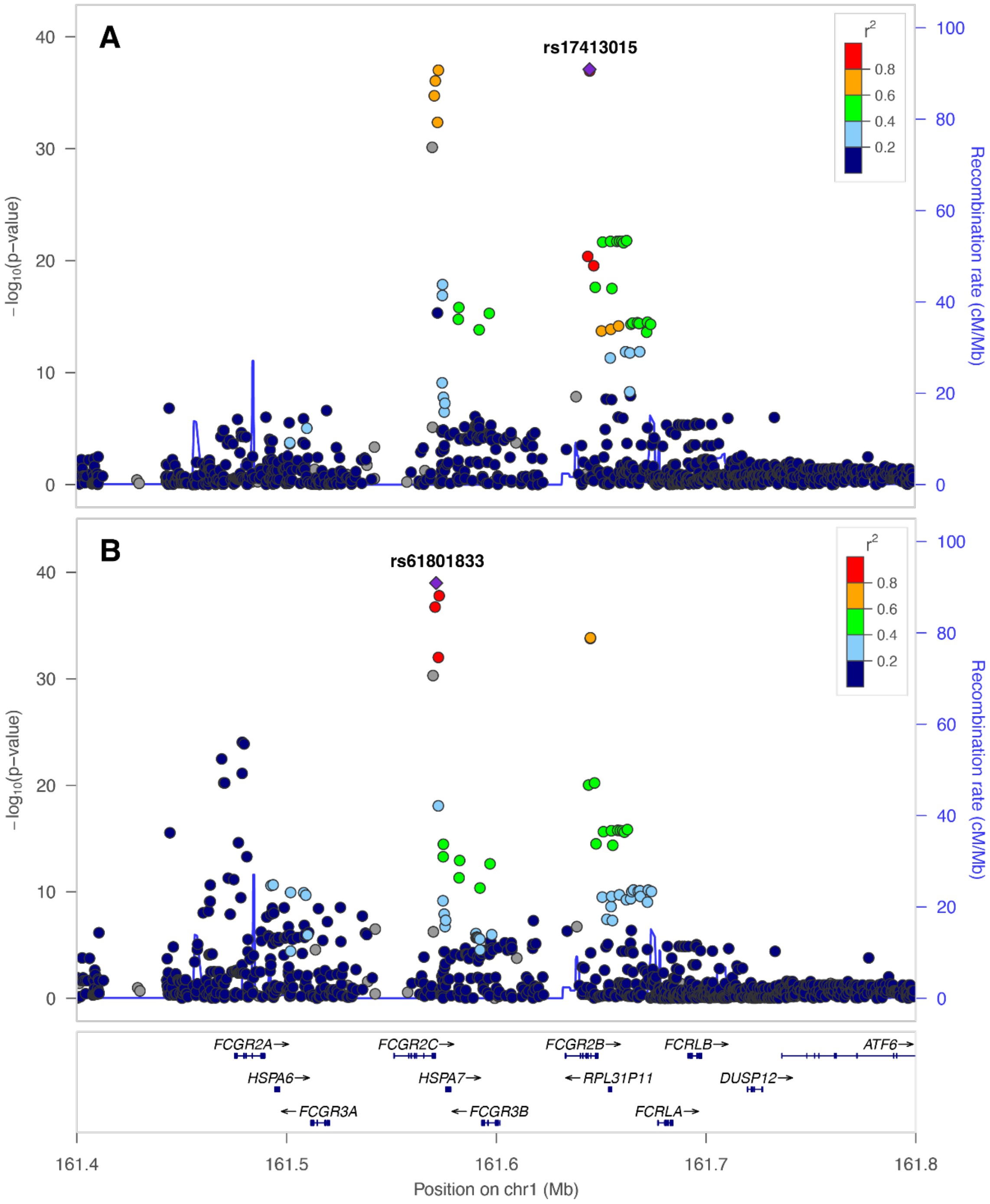

Supplementary Figure 2. Aptamer-Specific Differences in FCGR2B Protein Measurement. The showcased LocusZoom plots underline the aptamer-specific differences in capturing the FCGR2B protein measurements, with additional *cis*-associations in the neighbouring FCGR2A and FCGR2C regions. This differential capture between the two aptamers targeting the same protein shows the lack of specificity in targeting proteins sharing amino acid sequence homology and could influence the resultant variation in protein measurements. A – Aptamer ID 22960-8, B – Aptamer ID 3310-62

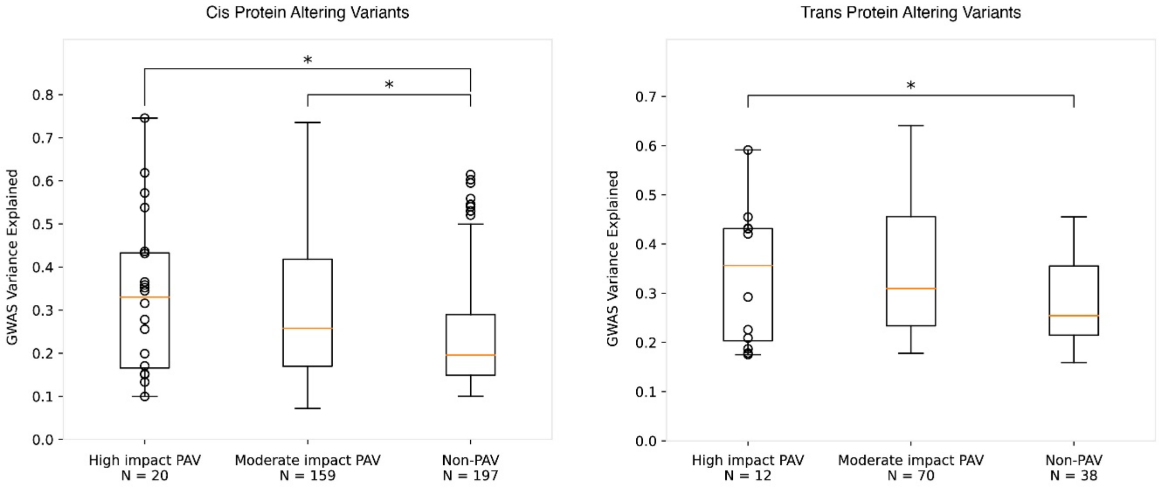

Supplementary Figure 3. Protein-Altering Variants (PAV) and Their Contribution to Protein Level Variance. These box plots show that SNPs with a higher impact on protein structure or expression tend to explain a greater variance in the associated protein concentrations compared to non-PAV SNPs. This trend is consistent in both *cis* and *trans* associations. The high-low impact and medium-low impact group differences were statistically significant ($p<0.05$) for *cis* associations. Meanwhile, for *trans* associations, only the high-low impact group comparison was statistically significant.

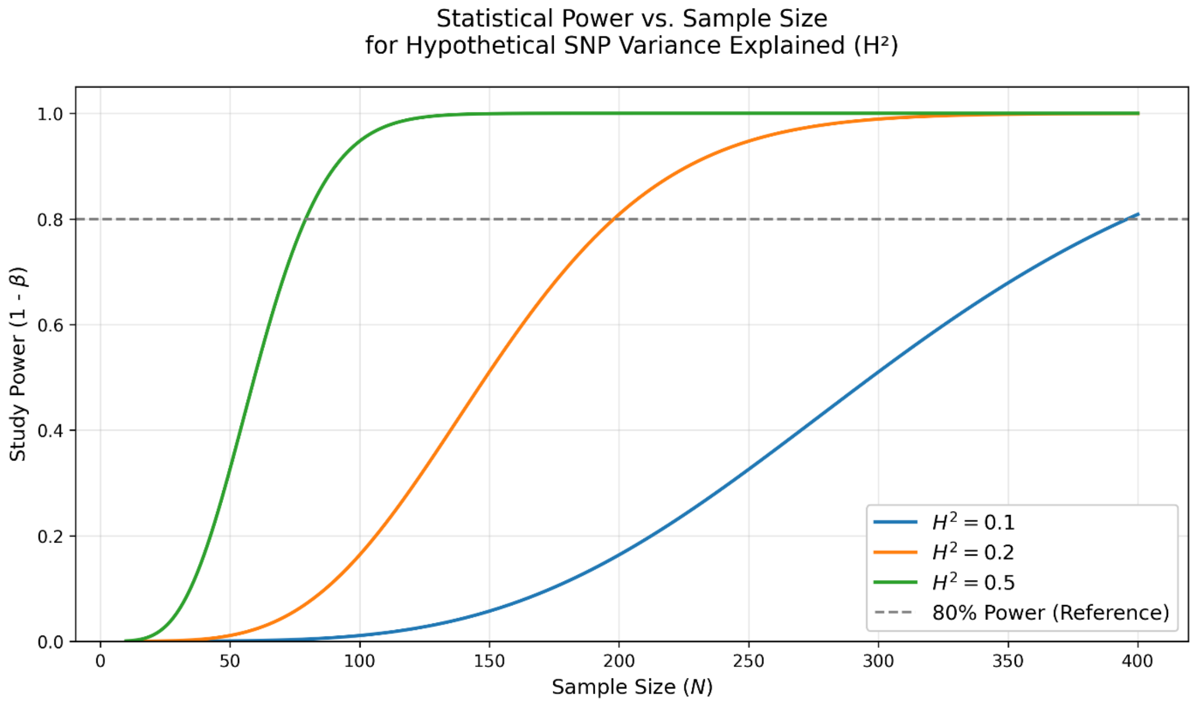

Supplementary Figure 4. Statistical power to detect pQTL of varying variance explained across sample sizes. The figure illustrates the statistical power of our study to detect genetic variants associated with protein levels given our sample size (N=200) and expected observed protein level variance explained magnitudes at Bonferroni-corrected genome-wide significance ($p = 5x10^{-8}$). The plot shows power curves calculated for different proportions of variance explained (H^2 = 10, 20, 50%), showing that at N=200 there is an >80% chance of detecting a H^2 = 20% genome-wide significant signal.

The power estimates incorporated three key components: First, the expected strength of the genetic signal was determined by multiplying the sample size by the assumed proportion of variance explained. Second, we established the critical χ^2 value corresponding to the genome-wide significance threshold with one degree of freedom. Finally, we computed the power as the probability that a test statistic from this non-central χ^2 distribution would exceed the critical threshold value. Power modelling was performed using python 3 with scipy.stats objects ncx2, chi2 (v1.9).

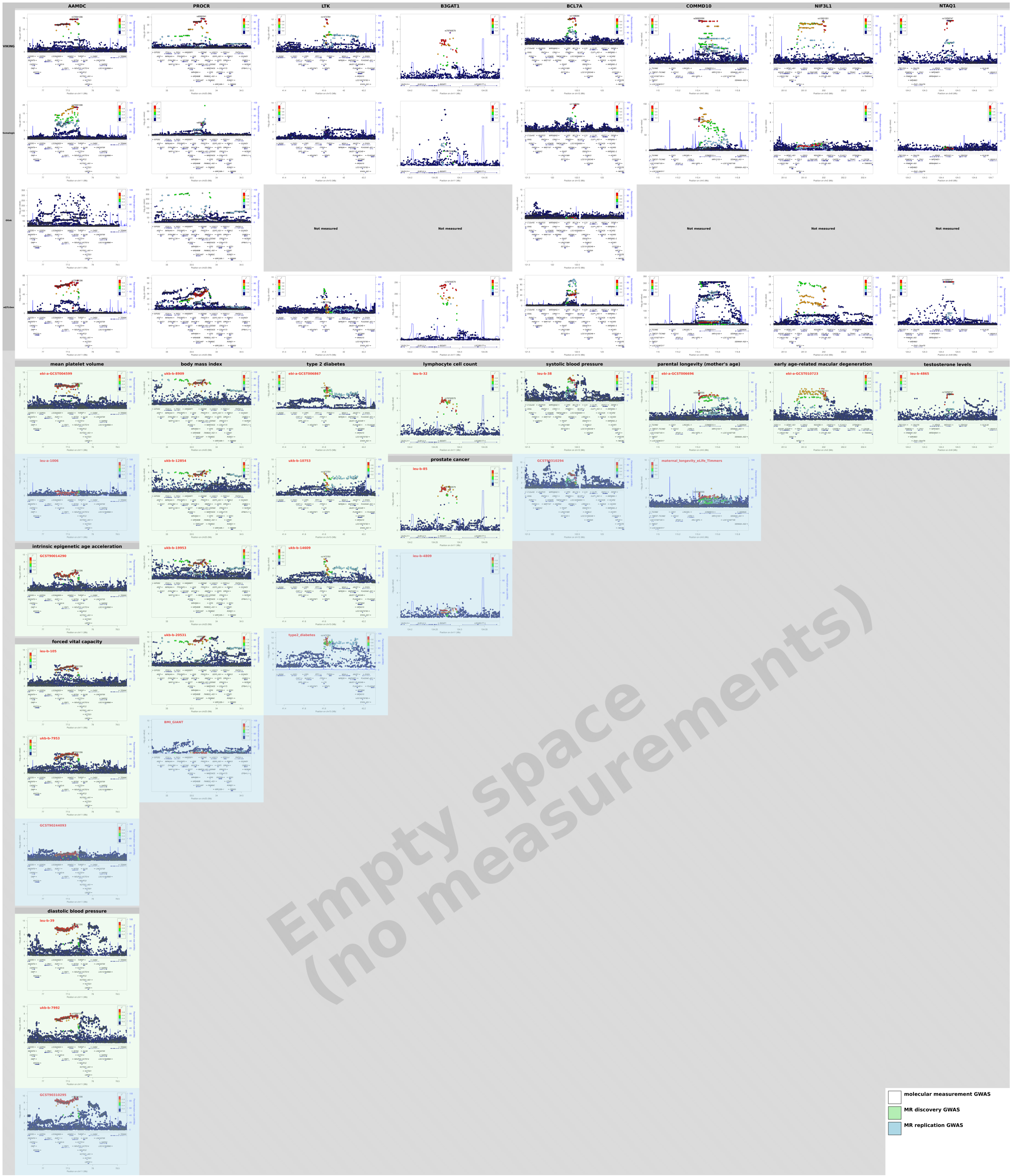

Supplementary Figure 5. LocusZoom Plots for Selected MR Instruments and Outcomes. This array of LocusZoom plots visualizes the genetic associations between selected MR exposures (top) and their corresponding outcomes (bottom), with each column representing one protein. The exposures used in this analysis come from three sources: SomaLogic - AASK (sample size 466, PMID: 35870639), Olink - UK Biobank (sample size 54,219, PMID: 37794186), and eQTLGen (sample size 31,684, PMID: 34475573). The exposure type is annotated on the top left side of the figure, while each outcome has an overlayed identification (ID), which can be referenced in Supplementary Table 8 for more detailed information on the studies. Outcomes are also split by colour into discovery and replication MR (see legend). The LD pattern in each LocusZoom plot is coloured after the sentinel SNP in the VIKING GWAS. The alignment of peaks across the exposure and outcome plots suggests that the associations are robust and likely reflect true genetic links between protein levels and disease outcomes.

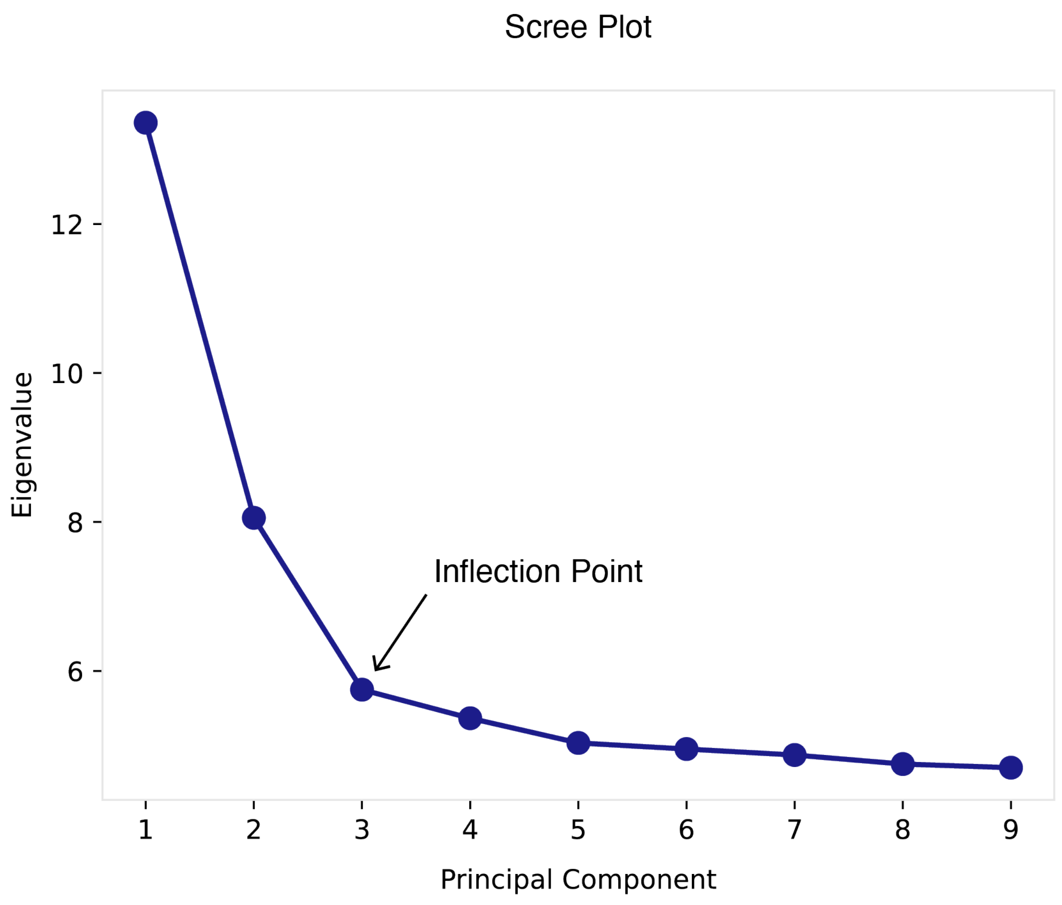

Supplementary Figure 6. Scree Plot Depicting the Relative Contribution of Principal Components to Total Variance. An extended VIKING cohort was used to extract the eigenvalues, comprising 2105, rather than just the 200 individuals with protein level measurements. The plot reveals a steep decline in eigenvalues, with an inflection point observed at PC3, as marked with an arrow. This inflection point delineates the threshold beyond which additional components contribute progressively less to explaining the total variance, underscoring the pivotal role played by the first three components in accounting for the genetic variability in the dataset. It encourages considering PC1, PC2, and PC3 as significant contributors in subsequent analyses, offering a balance between data reduction and retention of information.
