# Supplementary material for: Efficient candidate drug target discovery through proteogenomics in a Scottish cohort
Source: Commun Biol. 2025 Aug 29;8:1300. doi: 10.1038/s42003-025-08738-w (PMC12397405; doi:10.1038/s42003-025-08738-w)
Supplement: Supplementary file 3 — Supplementary Data descriptions [file 42003_2025_8738_MOESM3_ESM.docx]

Supplementary Data 1. Comprehensive Genomic and Proteomic Annotations of 505 Significant SNPs.

**HUGO**: The official symbol representing the gene according to the HUGO Gene Nomenclature Committee.

**Uniprot**: The identifier for the gene's corresponding protein entry in the Universal Protein resource database.

**SNP**: The rsid of the single nucleotide polymorphism being analysed in the study.

**Chromosome**: The chromosome number where the SNP of interest is located.

**Position**: The exact genomic location of the SNP on the chromosome based on the Genome Reference Consortium Human Build 37 (GRCh37).

**Individuals included in the study**: The number of individuals whose data passed the Quality Control steps and were included for GWAS of this protein measurement.

**Effect allele**: The allele associated with the change in studied protein level.

**Other allele**: The alternative allele present at the SNP locus not associated with the effect.

**Effect allele frequency in VIKING**: The frequency of the effect allele in the VIKING cohort.

**Effect allele freq in non-Finnish Eur**: The frequency of the effect allele in the non-Finnish European population (gnomAD).

**Effect Size (beta)**: The estimate of the effect size of the allele in terms of the change in studied protein level.

**Standard error of beta**: The standard error associated with the beta estimate.

**F-statistic**: Measure of the strength of the instrument if used for Mendelian Randomization, estimated as F = (R^2^/(1-R^2^)((n-k-1)/k), where R^2^ – Variance explained by the genetic variants, n – sample size, k – number of genetic instruments.

**P-value**: The statistical significance of the association between the SNP and the trait.

**Variance Explained**: The proportion of phenotypic variance explained by the SNP.

**Somamer targeted protein chromosome**: The chromosome where the protein targeted by the somamer is encoded.

**Somamer targeted protein TSS**: The transcription start site (TSS) of the gene encoding the somamer targeted protein (GRCh37).

**Bp distance between targeted protein TSS and SNP**: The distance in base pairs between the SNP and the TSS of the somamer targeted protein's gene.

**cisTrans**: Indicates whether the SNP effect is in cis (near the gene it affects, <1Mb) or in trans (affecting a gene located elsewhere, >1Mb).

**Overlapped Gene**: The gene that physically overlaps with the SNP locus.

**Nearest Upstream Gene**: The closest gene upstream to the SNP locus.

**Nearest Downstream Gene**: The closest gene downstream to the SNP locus.

**Most severe consequence**: The most critical biological effect associated with the SNP (Variant Effect Predictor, VEP).

**novel**: Indicates whether the measured protein has been present in previous large-scale proteomics panels.

**somamerID**: The identifier for the somamer used in the analysis.

**FlagQC**: Flags for quality control issues or concerns regarding the somamer, both by SomaLogic and inhouse methods.

**Replication p-value**: The statistical significance of the association between the SNP and the protein levels measured in Pietzner et.al, 2021 (PMID: 34648354). Association p-values originally reported as 0 in the referenced study, due to software limitations, are annotated here as 1x10^-300^, which reflects the lower threshold of the software's precision. Multiple values in the four replication columns separated by | represent multiple measurements of a particular protein with more than one aptamer.

**Replication Effect Size**: The estimate of the effect size of the allele in terms of the change in studied protein level in Pietzner et.al, 2021.

**Replication Allele Frequency**: The frequency of the effect allele in the Fenland cohort, studied in Pietzner et.al, 2021.

**Replication SomamerID**: The identifier for the somamer used in the replication analysis in Pietzner et.al, 2021.

Supplementary Data 2. VIKING *cis* pQTL replication in the AASK cohort study.

**HUGO**: The official symbol representing the gene according to the HUGO Gene Nomenclature Committee.

**VIKING rsid**: The rsid of the genome-wide significant single nucleotide polymorphism in this study.

**Phased Effect allele**: The allele associated with the change in studied protein level.

**Phased Other allele**: The alternative allele present at the SNP locus not associated with the effect.

**VIKING Effect Allele Frequency**: The frequency of the effect allele in the VIKING cohort.

**VIKING Effect Size**: The estimate of the effect size of the allele in terms of the change in studied protein level.

**VIKING p-value**: The statistical significance of the association between the SNP and the protein level in this study.

**VIKING SomamerID**: The identifier for the somamer used in the analysis in the VIKING study.

**Replication AASK rsid**: The rsid(s) of genome-wide significant single nucleotide polymorphisms from the AASK study (PMID 35870639). "NA" indicates that no SNP within LD (r² > 0.6) was found to match the rsid listed in the VIKING rsid field.

**Replication AASK LD**: Pairwise linkage disequilibrium between the sentinel SNP reported in VIKING and AASK. LD between the two of r^2^ >= 0.6 was considered to be originating from the same genetic signal.

**Replication AASK p-value**: The statistical significance of the association between the SNP and the protein level in the AASK study.

**Replication AASK Effect Size**: The estimate of the effect size of the allele in terms of the change in studied protein level in the AASK study.

**Replication AASK Effect Allele Frequency**: The frequency of the effect allele in the AASK cohort.

**Replication AASK SomamerID**: The identifier for the somamer(s) used in the analysis in the AASK study.

Supplementary Data 3. Catalogue of Proteins Assayed in Previous Large-Scale Proteomics Studies.

**HUGO or Other name**: Symbol representing the gene according to the HUGO Gene Nomenclature or another unclassified name extracted from supplementary large-scale proteomics studies.

**Uniprot**: The identifier for the gene's corresponding protein entry in the Universal Protein resource database.

Supplementary Data 4. Detailed Reference of Assayed Proteins with HUGO, Uniprot, and Sequence IDs.

**HUGO**: Symbol representing the gene according to the HUGO Gene Nomenclature

**Uniprot**: The identifier for the gene's corresponding protein entry in the Universal Protein resource database.

**Full Name**: The complete official name of the protein as recognized in biological and proteomic research and provided by SomaLogic.

**Entrez Gene Symbol**: The gene symbol as designated in the Entrez database.

**Sequence ID**: The unique Somamer identifier.

**Somamer ID**: Another unique Somamer identifier provided by Somalogic.

Supplementary Data 5. Compilation of Significant Bidirectional Mendelian Randomisation Findings.

**MR Exposure**: The HUGO name of the protein used in the Mendelian randomization study to observe its effect on the outcome.

**MR Outcome**: Name of the trait or condition investigated in the Mendelian randomization study.

**Study ID**: The unique identifier assigned to each individual study in OpenGWAS database.

**MR Method**: The specific method utilised in the Mendelian randomization analysis.

**MR Number of SNPs**: The total count of single nucleotide polymorphisms used in the Mendelian Randomisation analysis.

**MR Instrumental variable F-statistic**: Measure of the strength of the instrument if used for Mendelian Randomization, estimated as F = (R^2^/(1-R^2^)((n-k-1)/k), where R^2^ – Variance explained by the genetic variants, n – sample size, k – number of genetic instruments.

**MR Effect size (beta)**: The magnitude of the effect that the exposure has on the outcome in the Mendelian Randomisation analysis.

**MR Standard Error of beta**: The standard error associated with the effect size, representing uncertainty in the estimate.

**MR p-value**: The p-value in the Mendelian Randomisation analysis, indicating the statistical significance of the observed effect size.

**Reverse MR Number of SNPs**: The total count of single nucleotide polymorphisms used in the reverse Mendelian Randomisation analysis.

**Reverse MR Effect size (beta)**: The magnitude of the effect observed in the reverse Mendelian Randomisation analysis.

**Reverse MR Standard Error of beta**: The standard error associated with the effect size in the reverse Mendelian Randomisation analysis, representing uncertainty in the estimate.

**Reverse MR p-value**: The p-value in the reverse MR analysis, denoting the statistical significance of the observed effect size.

**Reverse MR Method**: The specific method utilised in the reverse Mendelian randomization analysis.

**Exposure and Outcome colocalises**: Evaluation on whether the exposure and outcome colocalise (Supplementary Data 7).

**MR replicates**: Evaluation on whether MR replicates with an independent instrument (Supplementary Data 10). An MR result was considered replicated if it demonstrated a consistent direction of MR effect between the discovery and replication analyses and had statistically significant evidence of association between the exposure and the outcome.

**MR replication significant**: Annotation of whether the MR replication passes the Bonferroni-corrected significance threshold for both independent (Stage 1) and semi-independent (Stage 2) MR replications was p = 0.05 / (26 Stage 1 + 80 maximum Stage 2 tests) = 4.72x10⁻⁴.

**MR replication matching direction**: Annotation of whether the MR replication matches in effect direction with the discovery analysis in VIKING.

**MR replication type**: Annotation of the type of MR replication. Independent – both independent exposure and outcome; Discovery MR Outcome – independent exposure and the same outcome GWAS as used in discovery.

**MR replication instrument**: Lists the types of independent exposures that were successfully replicated in MR analyses. If replication was unsuccessful, the column includes all the exposures that were tested during the replication attempts, regardless of outcome.

**Instrument colocalises with VIKING**: Indicates whether the replication instrument(s) colocalise with the VIKING instrument. (Supplementary Data 9).

**MR replication comment**: Provides an explanation for why MR replication was not performed, if applicable.

Supplementary Data 6. Colocalization Analysis Results.

**HUGO**: The gene symbol identifying the protein measured in this study used in the colocalization analysis.

**Uniprot**: The unique identifier from the UniProt database for the protein measured in this study.

**somamerID**: The unique SomaLogic identifier for the SOMAmer (Slow Off-rate Modified Aptamer) used in the analysis to specifically bind to a particular protein.

**Outcome**: The specific trait or health condition that is being studied in colocalization analysis.

**Open GWAS Dataset ID**: The unique OpenGWAS identifier for this dataset.

**Open GWAS Sample size**: The number of samples (case + control) included in the specific open GWAS dataset employed in the analysis.

**Number of SNP**: Count of single nucleotide polymorphisms involved in the colocalization analysis.

**PP.H0.abf**: The posterior probability that none of the datasets have a signal (i.e., a significant association).

**PP.H1.abf**: The posterior probability that the first dataset has a signal, which is not shared with the other dataset.

**PP.H2.abf:** The posterior probability that the second dataset has a signal, which is not shared with the other dataset.

**PP.H3.abf**: The posterior probability that there are signals in both datasets, arising from two independent variants.

**PP.H4.abf**: The posterior probability that the datasets share the same signal, arising from the same causal variant.

Supplementary Data 7. Summary of Replication MR Results.

**Replication MR Exposure**: Genetic exposures used in the replication Mendelian Randomization (MR) analysis, specifying the gene name and the source of the instrument. Instrument sources were Somalogic - sample size 466, PMID: 35870639, Olink - sample size 54,219, PMID: 37794186, eQTLGen - sample size 31,684, PMID: 34475573

**Replication MR Stage 1 Outcome**: Independent GWAS outcome datasets that were used in the replication MR analysis. "NA" indicates cases where no suitable independent outcome study was available for replication.

**Replication MR Stage 1 Effect size (beta)**: The magnitude of the effect that the exposure has on the outcome in the independent MR replication analysis. "NA" indicates cases where no suitable independent outcome study was available for replication.

**Replication MR Stage 1 Standard Error of beta**: The standard error associated with the effect size, representing uncertainty in the estimate.

**Replication MR Stage 1 p-value**: The p-value in the MR independent replication analysis, indicating the statistical significance of the observed effect size.

**Replication MR Number of SNPs**: Count of single nucleotide polymorphisms used in the MR independent replication analysis.

**Replication MR Stage 2 effect size (beta)**: The magnitude of the effect that the independent exposure has on the discovery outcome GWAS used in the semi-independent MR replication analysis. "NA" indicates cases where independent replication was successful.

**Replication MR Stage 2 beta standard error**: The standard error associated with the effect size, representing uncertainty in the estimate.

**Replication MR Stage 2 p-value**: The p-value in the MR semi-independent replication analysis, indicating the statistical significance of the observed effect size.

**Replication MR Stage 2 Number of SNPs**: Count of single nucleotide polymorphisms used in the MR semi-independent replication analysis.

**Discovery MR Outcome**: Trait analysed and found to be MR significant in the discovery MR analysis (Supplementary Data 6).

**Discovery MR Effect Size (beta)**: The magnitude of the effect that VIKING exposure has on the discovery outcome GWAS in MR analysis.

**Discovery MR p-value**: The p-value in the VIKING-outcome discovery MR analysis, indicating the statistical significance of the observed effect size.

**Replication MR Stage 1 Study ID**: The unique identifier for the independent outcome dataset. "NA" indicates cases where no suitable independent outcome study was available for replication.

**Replication MR Stage 1 Method**: The specific method utilised in the MR independent replication analysis.

**Replication MR Stage 1 Instrumental variable F-statistic**: Measure of the strength of the instrument used for Mendelian Randomization independent replication, estimated as F = (R^2^/(1-R^2^)((n-k-1)/k), where R^2^ – Variance explained by the genetic variants, n – sample size, k – number of genetic instruments.

**Replication Stage 1 Outcome Study Author**: First author of the GWAS study used as outcome in independent MR replication.

**Replication Stage 1 Outcome Study Publication Year**: Publication year of the GWAS study used as outcome in independent MR replication.

**Replication Stage 1 Outcome Study Sample Size**: Sample size of the GWAS study used as outcome in independent MR replication.

**Replication Stage 1 Outcome Study pmid**: PMID of the GWAS study used as outcome in independent MR replication.

**Replication MR Stage 2 Method**: The specific method utilised in the MR semi-independent replication analysis. "NA" indicates cases where independent replication was successful.

**Replication MR Stage 2 Instrumental variable F-statistic**: Measure of the strength of the instrument used for Mendelian Randomization semi-independent replication, estimated as F = (R^2^/(1-R^2^)((n-k-1)/k), where R^2^ – Variance explained by the genetic variants, n – sample size, k – number of genetic instruments.

**Discovery MR Study ID**: The unique identifier assigned to each individual outcome study in OpenGWAS database used in discovery MR. These studies were also used as outcomes in semi-independent MR replication analysis.

**Discovery MR Outcome Study Publication Year**: Publication year of the GWAS study used as outcome in discovery MR and MR semi-independent replication.

**Discovery MR Outcome Study Sample Size**: Sample size of the GWAS study used as outcome in discovery MR and MR semi-independent replication.

**Discovery MR Outcome Study pmid**: PMID of the GWAS study used as outcome in discovery MR and MR semi-independent replication.

**MR Significant result**: Annotation of whether the MR replication found a significant exposure-outcome link. The Bonferroni-corrected significance threshold for both independent and semi-independent MR replications was p = 0.05 / (26 fully independent + 80 maximum semi-independent tests) = 4.72x10⁻⁴

Supplementary Data 8. Colocalisation results between VIKING and other pQTL and eQTL datasets.

**Gene name**: The gene symbol identifying the protein or eQTL being colocalised in the analysis.

**somamerID**: Unique SomaLogic identifier for the SOMAmer (Slow Off-rate Modified Aptamer) used in the analysis to specifically bind to a particular protein.

**comparison assay**: One of three protein or gene expression studies being compared against. somalogic - sample size 466, PMID: 35870639, olink - sample size 54,219, PMID: 37794186, eQTLGen - sample size 31,684, PMID: 34475573.

**comparison region max -logp**: Most significant association by p-value within 300kb of the VIKING sentinel SNP (Supplementary Data 1).

**nsnps**: Count of single nucleotide polymorphisms involved in the colocalization analysis.

**H0**: The posterior probability that the first dataset has a signal, which is not shared with the other dataset.

**H1**: The posterior probability that the second dataset has a signal, which is not shared with the other dataset.

**H2**: The posterior probability that the second dataset has a signal, which is not shared with the other dataset.

**H3**: The posterior probability that there are signals in both datasets, arising from two independent variants.

**H4**: The posterior probability that the datasets share the same signal, arising from the same causal variant.

Supplementary Data 9**.** Gene enrichment analysis results.

**Gene Ontology Term**: Descriptions of Gene Ontology (GO) terms, each accompanied by its specific GO identifier. A combination of associated biological processes, molecular functions, and cellular components, providing an overview of the functional attributes of the genes in the analysed dataset.

**Reference Gene Count**: Number of genes associated with each GO term within the total reference 20592 Homo sapiens gene set.

**Protein match**: Number of uploaded proteins that link to a specific GO term.

**Expected Protein Count**: Number of proteins from the uploaded set that would be associated with each GO term under a random distribution.

**Enrichment Direction**: Indicates the direction of enrichment for each GO term in the uploaded dataset. A '+' denotes overrepresentation, meaning that the GO term is more prevalent in the dataset than expected based on the reference gene set. Conversely, a '-' signifies underrepresentation.

**Fold Enrichment**: Ratio that compares the proportion of a specific GO term in the dataset to its proportion in the reference gene set.

**Raw P-value**: Raw p-values for each GO term in relation to the uploaded dataset, as calculated from Fisher's Exact Test.

**FDR**: False Discovery Rate (FDR) values for each GO term in relation to the uploaded dataset, providing an estimate of the expected proportion of false positives.

Supplementary Data 10. Drug target annotation for the clinically relevant findings identified in this study.

**Exposure**: The protein target identified in our study as potentially causally linked to medical traits through Mendelian Randomization analysis.

**Linked Outcomes through MR**: The disease or trait phenotypes significantly associated with the protein exposure in Mendelian Randomization.

**Drugbank link**: Link to the DrugBank database entry for the protein target, when present.

**Drugbank drug entries**: The total number of Approved or Investigational drugs in DrugBank for the protein target.

**Drugbank Medical indications**: Current clinical uses of the listed drugs (if any) for each target in Drugbank.

**Drugbank adverse effects & toxicity**: Documented side effects, contraindications, or safety concerns for drugs targeting this protein.

**ChEMBL link**: Link to the ChEMBL database entry for the protein target.

**ChEMBL drug entries**: The total number of Approved or Investigational drugs in ChEMBL for the protein target.

**ChEMBL Medical Indications**: Current clinical uses of the listed drugs (if any) for each target in ChEMBL.

Supplementary Data 11. VIKING sub cohort characteristic summary split by sex.

**Sex**: Indicates the fraction of the biological sex of the individual (male or female) in the population subset analysed in this study.

**Age**: The mean age of the participant in years.

**Education Level, years**: The mean total number of years of formal education completed.

**Diabetic**: The fraction of study participants that have been diagnosed with diabetes.

**Body Mass Index (BMI)**: The mean measure of body fat based on weight and height (kg/m²).

**Systolic Blood Pressure**: The mean pressure in the arteries during the contraction of the heart muscle (mmHg).

**Diastolic Blood Pressure**: The mean pressure in the arteries when the heart is at rest between beats (mmHg).

**Blood Pressure Medicated**: The fraction of study participants that are taking medication to control blood pressure.

**Forced Expiratory Volume (1 sec) (fev1)**: The mean volume of air exhaled during the first second of a forced breath (liters).

**Total Cholesterol**: The mean of total amount of cholesterol in the blood (mg/dL).

Supplementary Data 12. Determining the Medical Relevance of Various Traits and Diseases with a Large Language Model.

**Outcome**: OpenGWAS dataset (ieu-a, ieu-b, ebi-a, ukb-b) outcome names that were used as input for categorisation.

**ChatGPT Pass1-5**: The ChatGPT assignment of whether the trait is medically actionable in each of the 5 passes.

**Prompt**: The prompt used for as the start of input for each of the passes.

Supplementary Data 13. Annotated Compilation of openGWAS Datasets.

**id.outcome:** The unique identifier assigned to the particular outcome or endpoint being studied in the dataset.

**trait:** The specific trait or characteristic that is the focus of the study.

**population:** The demographic or ethnic group that the study participants belong to.

**sample_size:** The total number of individuals who participated in the study.

**ncase:** The number of individuals in the study who have the condition or trait being investigated.

**ncontrol:** The number of individuals in the study serving as a control group, without the condition or trait being investigated.

**subcategory:** The separation of outcomes into Binary or Continuous phenotypes.

**consortium**: The name of the consortium or group that carried out the study.

**author**: The primary author or investigator responsible for the study.

**year**: The year the study was conducted or published.

**pmid**: The PubMed identifier for the study.

**outcome**_**name**: The converted name given to the outcome or endpoint studied for ChatGPT annotation.

**Clinically relevant**: A column indicating whether the outcome or trait has significant implications or applications in a clinical setting as assessed with ChatGPT.

Supplementary Data 14. Colocalization analysis between independent replication instruments and outcomes.

**Gene name**: The gene symbol identifying the protein measured in this study used in the colocalization analysis.

**exposure**: One of three protein or gene expression studies used as independent instruments. Somalogic - sample size 466, PMID: 35870639, Olink - sample size 54,219, PMID: 37794186, eQTLGen - sample size 31,684, PMID: 34475573.

**nsnps**: Count of single nucleotide polymorphisms involved in the colocalization analysis.

**H0**: The posterior probability that the first dataset has a signal, which is not shared with the other dataset.

**H1**: The posterior probability that the second dataset has a signal, which is not shared with the other dataset.

**H2**: The posterior probability that the second dataset has a signal, which is not shared with the other dataset.

**H3**: The posterior probability that there are signals in both datasets, arising from two independent variants.

**H4**: The posterior probability that the datasets share the same signal, arising from the same causal variant.

**MR significant**: Annotation of whether the link between exposure and outcome was found to be MR significant (Supplementary Data 11).
