# Supplementary material for: Efficient candidate drug target discovery through proteogenomics in a Scottish cohort
Source: Commun Biol. 2025 Aug 29;8:1300. doi: 10.1038/s42003-025-08738-w (PMC12397405; doi:10.1038/s42003-025-08738-w)
Supplement: Supplementary file 5 — Reporting Summary [file 42003_2025_8738_MOESM5_ESM.pdf]

nature portfolio

Corresponding author(s): James F. Wilson

Last updated by author(s): 2025-08-05

Reporting Summary

Nature Portfolio wishes to improve the reproducibility of the work that we publish. This form provides structure for consistency and transparency in reporting. For further information on Nature Portfolio policies, see our Editorial Policies and the Editorial Policy Checklist.

Statistics

For all statistical analyses, confirm that the following items are present in the figure legend, table legend, main text, or Methods section.

n/a	Confirmed	
[ ]	[X]	The exact sample size (*n*) for each experimental group/condition, given as a discrete number and unit of measurement
[ ]	[X]	A statement on whether measurements were taken from distinct samples or whether the same sample was measured repeatedly
[ ]	[X]	The statistical test(s) used AND whether they are one- or two-sided<br>*Only common tests should be described solely by name; describe more complex techniques in the Methods section.*
[ ]	[X]	A description of all covariates tested
[ ]	[X]	A description of any assumptions or corrections, such as tests of normality and adjustment for multiple comparisons
[ ]	[X]	A full description of the statistical parameters including central tendency (e.g. means) or other basic estimates (e.g. regression coefficient) AND variation (e.g. standard deviation) or associated estimates of uncertainty (e.g. confidence intervals)
[ ]	[X]	For null hypothesis testing, the test statistic (e.g. *F*, *t*, *r*) with confidence intervals, effect sizes, degrees of freedom and *P* value noted<br>*Give P values as exact values whenever suitable.*
[X]	[ ]	For Bayesian analysis, information on the choice of priors and Markov chain Monte Carlo settings
[X]	[ ]	For hierarchical and complex designs, identification of the appropriate level for tests and full reporting of outcomes
[ ]	[X]	Estimates of effect sizes (e.g. Cohen's *d*, Pearson's *r*), indicating how they were calculated

Our web collection on statistics for biologists contains articles on many of the points above.

Software and code

Policy information about availability of computer code

Data collection	No Software was used.
Data analysis	RegScan v0.5; PANTHER 19.0; PLINK 1.9; LDproxy (ver. August 2022); ChatGPT 4 (ver. July 20); clustalo v1.2.4; Python3: pandas 1.4; scipy 1.4; numpy 1.20; requests 2.22; matplotlib 3.2; R 3.6.3 & 4.1.0: TwoSampleMR 0.5.6; coloc 5.1.0.

For manuscripts utilizing custom algorithms or software that are central to the research but not yet described in published literature, software must be made available to editors and reviewers. We strongly encourage code deposition in a community repository (e.g. GitHub). See the Nature Portfolio guidelines for submitting code & software for further information.

Data

Policy information about availability of data

All manuscripts must include a data availability statement. This statement should provide the following information, where applicable:

- Accession codes, unique identifiers, or web links for publicly available datasets
- A description of any restrictions on data availability
- For clinical datasets or third party data, please ensure that the statement adheres to our policy

The summary association statistics for all proteomic GWAS in this study have been deposited to the GWAS Catalog (https://www.ebi.ac.uk/gwas/, Study Accession IDs GCST90436912 – GCST90444200). There is neither Research Ethics Committee approval, nor consent from individual participants, to permit open release of the individual-level research data underlying this study. The datasets generated and analysed during the current study are therefore not publicly available. Instead, the research data and/or DNA samples are available by managed access from accessQTL@ed.ac.uk, following approval by the QTL Data Access Committee and in line [illegible]

with the consent given by participants. Each approved project is subject to a data or materials transfer agreement (D/MTA) or commercial contract. The UK Biobank genotypic data used in this study as a LD reference panel were approved under application 19655 and are available to qualified researchers via the UK Biobank data access process.

Research involving human participants, their data, or biological material

Policy information about studies with human participants or human data. See also policy information about sex, gender (identity/presentation), and sexual orientation and race, ethnicity and racism.

Reporting on sex and gender	Sex data was collected, analysed, and appropriately referenced in this study. Where applicable, statistical tests were conducted to assess sex-related effects. No gender-related analyses were performed, as gender was not a variable in the datasets.
Reporting on race, ethnicity, or other socially relevant groupings	The study utilized a geographically defined cohort, which consists of individuals with all four grandparents originating from the Shetland Isles. Participants self-reported their ancestry through a health survey questionnaire. No other socially constructed or relevant categorization variables, such as race or ethnicity, were collected. Confounding variables, such as age and kinship, were accounted for in the analysis to minimize potential biases.
Population characteristics	See above.
Recruitment	Recruitment in Viking Genes is described in Kerr et al (2019) Scientific Reports 9:10964.
Ethics oversight	All participants in the Viking Health Study—Shetland (VIKING) gave written informed consent for broad ranging health and ancestry research including, whole genome/exome sequencing and the study was given a favourable opinion by the South East Scotland Research Ethics Committee (REC Ref 12/SS/0151). All Viking Genes participants are now unified under South East Scotland REC reference: 19/SS/0104.

Note that full information on the approval of the study protocol must also be provided in the manuscript.

Field-specific reporting

Please select the one below that is the best fit for your research. If you are not sure, read the appropriate sections before making your selection.

☒ Life sciences ☐ Behavioural & social sciences ☐ Ecological, evolutionary & environmental sciences

For a reference copy of the document with all sections, see nature.com/documents/nr-reporting-summary-flat.pdf

Life sciences study design

All studies must disclose on these points even when the disclosure is negative.

Sample size	No formal power calculation was performed. In quantitative genetics, the larger the sample size, the greater the ability to investigate rarer alleles, for there are more observations of each allele. The sample sizes here were constrained by the availability of SomaLogic proteomic data A sample size of 200 was used in this study, results of which have been replicated in other studies of sample sizes exceeding 10,000.
Data exclusions	Protein abundances were filtered by removing outliers beyond three interquartile ranges from the median. Only common (allele frequency higher than 0.05) genetic variants were analysed. All these exclusion criteria were pre-established.
Replication	cis pQTL replication was conducted using GWAS summary statistics from Pietzner et al., 2021, achieving full possible effect size and it's directionality replication in the overlapping protein targets.<br>Mendelian Randomisation analyses were replicated using 3 independent pQTL and one eQTL dataset and multiple outcome GWAS, confirming 26/39 associations. Non-replicated associations (10/13) were primarily related to blood cell counts, likely reflecting cohort heterogeneity, statistical power differences, or technical variability across platforms (SomaScan, Olink, eQTL).
Randomization	The present study does not describe a randomised controlled trial, thus allocation and randomisation are not relevant.
Blinding	The present study does not describe a blinded randomised controlled trial, thus it was not necessary to blind researchers. Subjects analysed in this study were volunteers from the general population. Allocation, treatment and randomisation are not relevant to this study.

Reporting for specific materials, systems and methods

We require information from authors about some types of materials, experimental systems and methods used in many studies. Here, indicate whether each material, system or method listed is relevant to your study. If you are not sure if a list item applies to your research, read the appropriate section before selecting a response.

Materials & experimental systems

n/a	Involved in the study	
☒	☐	Antibodies
☒	☐	Eukaryotic cell lines
☒	☐	Palaeontology and archaeology
☒	☐	Animals and other organisms
☒	☐	Clinical data
☒	☐	Dual use research of concern
☒	☐	Plants

Methods

n/a	Involved in the study	
☒	☐	ChIP-seq
☒	☐	Flow cytometry
☒	☐	MRI-based neuroimaging

Plants

Seed stocks	NA
Novel plant genotypes	NA
Authentication	NA
